# Modeling of Processing-Induced Pore Morphology in an Additively-Manufactured Ti-6Al-4V Alloy

**DOI:** 10.3390/ma10020145

**Published:** 2017-02-08

**Authors:** Mohammad Rizviul Kabir, Henning Richter

**Affiliations:** German Aerospace Center, Institute of Materials Research, Linder Höhe, 51147 Köln, Germany; henning.richter@dlr.de

**Keywords:** selective laser melting (SLM), pore morphology, microstructure modeling, Voronoi tessellations, finite-element-method (FEM)

## Abstract

A selective laser melting (SLM)-based, additively-manufactured Ti-6Al-4V alloy is prone to the accumulation of undesirable defects during layer-by-layer material build-up. Defects in the form of complex-shaped pores are one of the critical issues that need to be considered during the processing of this alloy. Depending on the process parameters, pores with concave or convex boundaries may occur. To exploit the full potential of additively-manufactured Ti-6Al-4V, the interdependency between the process parameters, pore morphology, and resultant mechanical properties, needs to be understood. By incorporating morphological details into numerical models for micromechanical analyses, an in-depth understanding of how these pores interact with the Ti-6Al-4V microstructure can be gained. However, available models for pore analysis lack a realistic description of both the Ti-6Al-4V grain microstructure, and the pore geometry. To overcome this, we propose a comprehensive approach for modeling and discretizing pores with complex geometry, situated in a polycrystalline microstructure. In this approach, the polycrystalline microstructure is modeled by means of Voronoi tessellations, and the complex pore geometry is approximated by strategically combining overlapping spheres of varied sizes. The proposed approach provides an elegant way to model the microstructure of SLM-processed Ti-6Al-4V containing pores or crack-like voids, and makes it possible to investigate the relationship between process parameters, pore morphology, and resultant mechanical properties in a finite-element-based simulation framework.

## 1. Introduction

Already being a well-established manufacturing process in the production of polymer-based components, Selective laser melting (SLM) of metal powders for producing near net-shape components has recently gained increased attention. The identification of suitable alloys and raw powders, and the optimization of the process parameters to obtain defect-free, high-strength components with desired mechanical properties, are topics of active research [1]. Recent activities have focused on the processing of titanium alloys, such as Ti-6Al-4V, which is used for the fabrication of components with complex geometry, as required in biomedical and aerospace applications.

With regard to the Ti-6Al-4V alloy, the SLM process parameters and thermal history are known to significantly affect microstructure formation [2,3,4]. Thermal post-treatment may cause phase transformations and variations in grain morphology [5], while improper process parameters result in voids and crack-like defects showing complex geometrical features [4]. The microstructure, particularly voids and defects, governs the effective mechanical properties of the manufactured components, such as tensile or fatigue strength [6,7,8,9]. A detailed understanding of the interdependency between the process parameters, microstructure, and mechanical properties, is therefore required, in order to be able to predict and optimize the mechanical performance of components processed by SLM.

A number of studies have investigated the interdependency between the process parameters, microstructure, and mechanical properties, via experimental means [10,11,12,13]. Alternatively, micromechanics-based numerical simulation approaches may provide an adaptable and cost-effective means to advance the current understanding of this interdependency. However, such approaches are less well represented in the literature, mainly due to the difficulty in modeling the microstructure of an SLM-processed Ti-6Al-4V alloy, which consists of elongated grains with fine acicular phases and pores with complex geometry. Models for micromechanics-based numerical simulations have to be sufficiently detailed to incorporate the relevant characteristics of the complex Ti-6Al-4V microstructure resulting from the SLM process, yet model generation has to be efficient and flexible enough to account for significant microstructure variations due to altered process parameters. In addition, model generation should facilitate the use of statistical information derived from microstructural investigations of the actual alloy.

Published works on micromechanics-based numerical simulation approaches mainly focus on the modeling of defect morphologies, such as pores or crack-like defects, considering the surrounding bulk material as a homogeneous domain [14,15,16]. Other works deliberately model the polycrystalline material with detailed crystallographic and grain morphological features, without considering the presence of pores or crack-like defects, see [17,18] and references therein. However, a fair description of the microstructural features, comprising both the grain morphology and the embedded pores with intricate geometric details, has not been attempted in these approaches.

In the present paper, a comprehensive modeling strategy for the SLM-fabricated microstructures of a Ti-6Al-4V alloy is proposed. Two essential aspects of this approach are, firstly, the independent creation of separate models for the microstructural features that critically influence the overall mechanical properties; and, secondly, the combination of these separate models, using a mesh superposition technique to reconstruct the microstructure of SLM-processed Ti-6Al-4V. During the first step, software tools, either self-written or open-source, are used to generate independent models for particular microstructural features. For instance, the grain microstructure is modeled by means of Voronoi tessellations, and the complex pore geometry is recreated by strategically arranging the overlapping spheres, using a self-written script. In the second step, these independent models are discretized with equal-sized voxel elements, and a mesh superposition technique is applied to obtain the final microstructure model.

The applicability of the proposed modeling strategy is demonstrated for two distinct pore geometries: spherical pores with convex boundaries, resulting from excessive laser energy density during the SLM process; and elongated, crack-like pores with concave boundaries, caused by a lack of laser energy density.

The microstructure and defect patterns of the Ti-6Al-4V alloy produced by other metal additive manufacturing processes, e.g., Electron beam melting (EBM) [19,20,21], can be analogously modeled using the suggested modeling strategy.

## 2. Materials and Methods

### 2.1. Microstructure

Ti-6Al-4V processed by SLM exhibits a characteristic polycrystalline microstructure and distinct pore geometries. The polycrystalline microstructure, with elongated grains which consist of lamellar-like, very fine *α*’-hexagonal-close-packed (hcp) phases [2,22,23], is the result of highly localized heating of the raw powder by the laser beam and layer-by-layer material build-up. During each subsequent pass of the laser beam, previous layers are locally re-melted, causing solidification and grain growth along the building direction. Reversions of the scanning direction lead to alternating grain directions showing a herringbone pattern [2]. Furthermore, due to the local heat transfer condition during processing, the microstructure varies along the building direction.

The as-built microstructure is not completely defect-free. Defects in the form of pores and crack-like voids occur during the melt-pool formation and after solidification of the melted particles. Details of the processing-induced pores, and the correlation between the process parameters and porosity, are described in the following section, in order to provide basic information regarding the proposed modeling strategy.

### 2.2. Processing-Induced Porosity

In the manufacturing of the Ti-6Al-4V alloy using SLM, the pore formation during layer-by-layer material build-up depends on both the quality of the raw powder, and the process parameters.

Powder quality is influenced by the chemical composition, powder morphology, and powder size distribution. For powders with a particular chemical composition, good powder flowability and high packing density of the powder bed can be obtained by using raw powders with spherical particles, and a particle size distribution such that 90% of the particles exhibit a size below 50 µm and particle sizes less than 10 µm are avoided [24]. The presence of smaller particles is required to minimize inter-particle voids and to obtain good packing quality for parts with a higher density [25]. However, very small particles may increase the inter-particle friction, resulting in poor powder flowability [26]. Poor flowability of the raw powder and low packing density influence the thermophysical characteristics of the powder bed. As a result, locally varied heat-affected zones may occur, leading to a defective microstructure containing a considerable number of irregularities, such as pores, voids, and gaps between unmelted powder particles.

Other major causes of defects or local irregularities are improper process parameters. For Ti-6Al-4V powders, the process parameters that crucially influence the formation of pores are the laser power *P*, scanning velocity *v*, hatch distance *h*, and laser focus *F*. Besides, parameters such as the layer thickness *δ*, build-platform heating, or scanning sequence, need to be adjusted in order to minimize pore formation.

A detailed analysis of pore formation, with respect to the variation of the process parameters *P*, *v*, *h*, and *δ*, was conducted by Kasperovich and co-workers [4,23]. All of these parameters can be related to the global energy density *E*, by:

*E* = *P*/(*v·δ·h*), (1)

which indicates that the energy density *E* can be used as a global variable to correlate process parameters and porosity.

**Figure 1:**
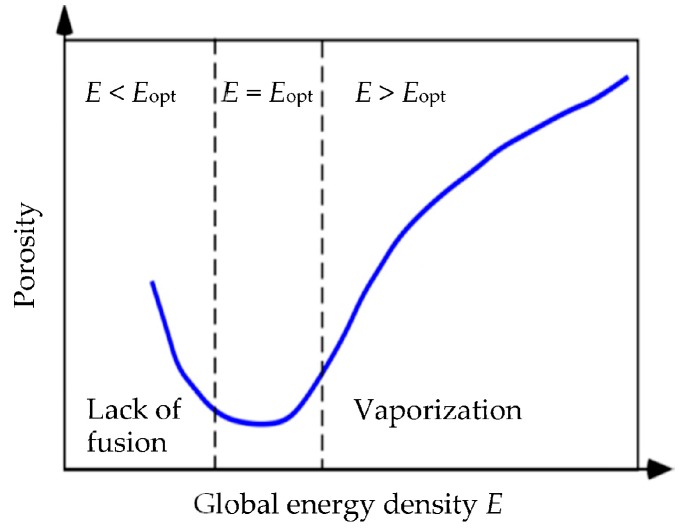
Effect of global energy density *E* on porosity in an SLM-processed Ti-6Al-4V alloy (schematic after [4,23]).

Figure 1 shows the relationship between global energy density and porosity for the SLM-processed Ti-6Al-4V alloy, as regenerated from the data presented in the literature [4,23]. The minimum of the curve indicates that a nearly pore-free microstructure can be obtained for an optimized energy density, *E*_opt_. However, for both *E* < *E*_opt_ and *E* > *E*_opt_, the volume content of defects in the form of pores, voids, and unprocessed particles increases, and the defect morphologies are different, as outlined in the following.

**Figure 2:**
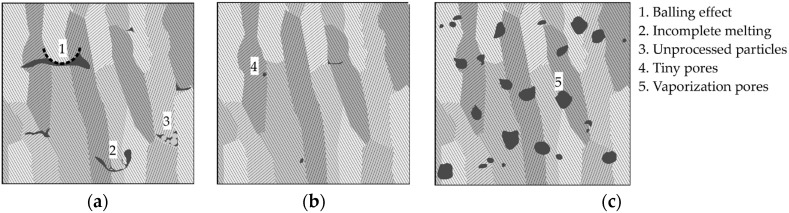
Schematic view of processing-induced pore morphology: (**a**) Lack of fusion pores due to low energy density; (**b**) Minimized porosity obtained for optimized energy density; (**c**) High energy vaporization pores with different sphericity.

At low energy density *E* < *E*_opt_, porosity increases, mainly due to the incomplete melting of the raw powder and the lack of fusion between powder particles, producing sharp, elongated, crack-like pores, as schematically shown in Figure 2a. In some cases, the melted material does not appropriately wet the underlying substrate, causing thermal mismatch across the melt pool. The melt pool experiences local surface tension and undergoes a break-up into segments, leading to the formation of elongated, crack-like pores upon solidification. This mechanism is known as balling effect [27]. In other cases, low energy density causes insufficiently melted powder particles, which are pulled into the melt pool. After the cooling of the melt pool, these particles agglomerate at the surface of the melt pool, producing hillocks. A lack of fusion due to low energy density may also result in unmelted powder particles becoming entrapped in the melt pool. In this case, pores form between and along the scanning tracks. Insufficient melting, combined with the balling effect and hillocks, as well as unprocessed particles, produces complex, elongated pores, with sharp, concave boundaries.

On the other hand, an excessive energy density *E* > *E*_opt_ causes vaporization of the material, creating spherical pores due to gas bubble formation [28,29,30]. As reported in the literature [31,32,33], high energy scan paths create deep, keyhole-type melt pools, which promote the formation of small pores. The pores associated with excessive energy density are predominantly spherical, with convex boundaries that are smaller than the pores occurring due to a lack of fusion (see Figure 2c). The spherical pores may exhibit circumferential, sharp rims at their surfaces, which are the result of the dynamic solidification of the melt pool.

Optimized process parameters result in almost defect-free microstructures, as exemplarily shown in Figure 2b.

### 2.3. Pore Geometry Characterization

In order to model the porosity caused by low or excessive global energy density, a detailed characterization of the processing-induced pore morphology and pore distribution occurring in actual specimens of SLM-processed Ti-6Al-4V has to be undertaken. Kasperovich et al. [4] classified the pores in the investigated Ti-6Al-4V specimens according to their sphericity *ψ*, which can be obtained by the relation:

(2) $$\psi \;=6V\sqrt{\frac{\pi }{A^{3}}},$$

where *A* and *V* are the surface area and the volume of the pore, respectively. Equation (2) describes the deviation of the pore geometry from an ideal sphere: for a sphere with surface area 4π *r*^2^ and volume (4π *r*^3^)/3, Equation (2) yields *ψ* = 1, whereas the sphericity of the actual pores in SLM-processed Ti-6Al-4V is characterized by 0 < *ψ* < 1. As estimated in [4], an insufficient global energy density during the SLM process leads to elongated pores with low sphericity (*ψ* < 0.7) and concave boundaries. With an excessive global energy density, the pores become more spherical (*ψ* > 0.7), and the pore boundaries are mostly convex.

The pore morphology can be further characterized by evaluating the principal curvatures of the pore surface. Pore size and orientation, with respect to the build direction, can be described in terms of aspect ratio, minimum and maximum Feret diameters, and Feret angles [4]. The spatial arrangement of multiple pores within the grain microstructure can be quantified with the aid of the mean inter-pore distance and nearest-neighbor distribution functions.

## 3. Modeling Strategy

### 3.1. Overview

To represent the actual microstructure of the SLM-processed Ti-6Al-4V alloy more accurately, numerical models have to incorporate the grain microstructure, the complex pore geometry, and variable pore orientation. With recent microanalytical investigation equipment and digital image processing software tools, detailed information about the complex microstructure can be obtained. As an example, computed microtomography (µ-CT) has been used for the visualization of the complex pore morphology in the SLM-processed Ti-6Al-4V alloy [4]. For other metallic alloys, three-dimensional reconstructions of pores have been generated, in order to derive models for digital image-based finite-element-analyses, elucidating the effects of these pores on the overall mechanical properties and the local stress distributions [14,16]. In these direct, digital image-based finite-element-analyses, the discretization of the complex geometries is one of the key challenges. For highly complex geometries, discretizing the domain with boundary conforming meshes is not straightforward, requiring time-consuming manual mesh repair efforts.

To avoid the complexity of µ-CT-based, real pore microstructure models, equivalent virtual pore models with parameterized geometries can be used. With the aid of mathematical algorithms, fed by statistical parameters derived from µ-CT, virtual microstructures with a realistic geometry can thus be created. The proposed comprehensive modeling strategy described in the following combines two different parameterized modeling approaches, to facilitate the implementation of the microstructural characteristics found in the SLM-processed Ti-6Al-4V alloy: the polycrystalline microstructure with elongated grains and acicular phases is modeled by means of a Voronoi tessellation; whereas pores with complex geometry, and either convex or concave boundaries, are approximated by strategically arranging (partly) overlapping spheres of varied sizes. The resulting models of the polycrystalline microstructure and of the pore space are generated separately and meshed with regular hexahedra, so-called voxel elements. The compatibility of the models is ensured by using meshes with an equal voxel size. Consequently, the accuracy of the geometry representation critically depends on the voxel size or the total number of voxels used.

To finally recreate the complex pore geometry within the grain microstructure, a mesh superposition technique is used, by which the corresponding voxel elements of the pore domain are removed from the mesh of the grain microstructure. Further details on the individual steps of this modeling strategy are given in the following sections.

### 3.2. Modeling of the Grain Microstructure

The grains contain fine, needle-shaped martensitic *α*’-hcp phases. The grain structure is elongated along the building direction, as described in Section 2.1. A model representation of this microstructure can be established by assuming a two-scale structure of the bulk material; at the coarse scale, the complete microstructure can be described by elongated grains, taking the length-to-width ratio from the analyses of real microstructures. Voronoi tessellations can be used to generate random-sized grains, which can be elongated to a desired aspect ratio in a later step. At the fine scale, the acicular martensite structure can be represented by fine lamellae. These lamellae can either be oriented arbitrarily, or arranged according to experimentally-known data.

In the present work, a well-established, Voronoi-based polycrystal generation software tool known as Neper [34] was used to generate the characteristic grain microstructure. However, additional script-controlled editing of the Voronoi seeds generated by Neper was required, in order to obtain the preferred microstructure. For the modeling of the acicular phases in the elongated grains, each grain was subdivided by fine lamellae with a particular orientation. Lamella thickness and orientation were defined by assigning a size parameter and a normal vector to the lamella plane. The self-written script basically controls which Voronoi algorithm is used, assigns a geometrical scaling factor to maintain the length/width ratio of the grains, and performs sub-structuring within the elongated grains, according to the given lamella thickness and orientation data.

**Figure 3:**
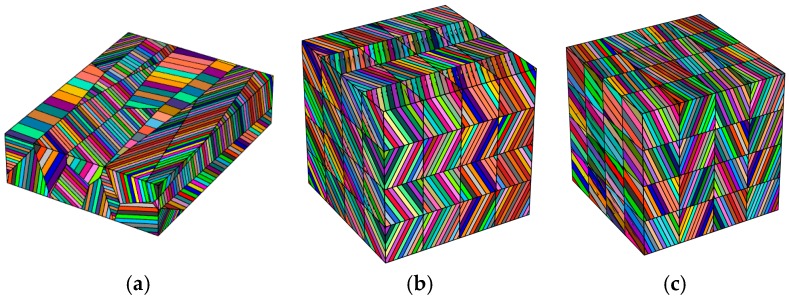
Exemplary grain microstructure models generated using Voronoi tessellation. The grains contain idealized, fine lamellae typical for acicular matensitic phases: (**a**) Polycrystal microstructure with elongated grains maintaining a length/width ratio > 2.5, with lamellar structure; (**b**) Idealized herringbone structure obtained for defined scanning strategy; (**c**) Oblique pattern assumed for arbitrary scanning strategy.

In Figure 3, exemplary virtual grain microstructures generated using Neper [34] are shown. Voronoi seed manipulation via the self-written script leads to realistic grain shapes with different arrangements of lamellae (Figure 3a). Moreover, arbitrary microstructure patterns obtained for different laser scanning strategies can be generated, e.g., herringbone (Figure 3b) or oblique patterns (Figure 3c).

### 3.3. Modeling of Pores with Convex Boundaries

**Figure 4:**
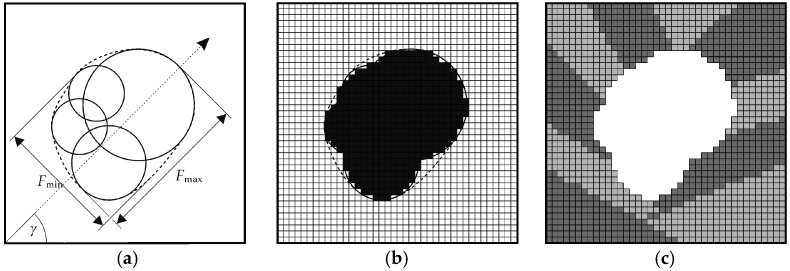
Modeling of pores with convex boundaries (schematic): (**a**) Approximation of actual pore geometry (dashed line) with Feret diameters, *F*_min_ and *F*_max_, and Feret angle *γ*, by placing overlapping spheres in a predefined bounding box; (**b**) Grid discretization of the sphere domain; (**c**) Elimination of the corresponding elements from the voxel mesh of the grain microstructure.

Complex pore geometries with convex boundaries can be approximated by strategically arranging overlapping spheres in a predefined bounding box, as schematically shown in Figure 4a for the two-dimensional case. The clustered arrangement of the spheres recreates the appearance of a spherical pore, resulting from an excessive laser energy density during SLM. By combining smaller and larger spheres, it is possible to account for fine ridges or grooves at the pore boundary. Furthermore, statistical information obtained by microstructural investigation techniques, such as scanning electron microscopy or computed microtomography, can be incorporated during model generation. As an example, Figure 4a shows the minimum and maximum Feret diameters (*F*_min_ and *F*_max_) of the pore geometry to be modeled. The latter is outlined by a dashed line. In addition, the orientation of the pore with respect to the bounding box can be arbitrarily chosen to correspond to the Feret angle *γ*. The sphericity *ψ* of the modeled pore can easily be determined based on the sum of the exterior voxel elements’ surface area.

The voxel discretization of the model is realized by generating an equidistant grid for the entire bounding box using a self-written script, see Figure 4b. Voxel elements representing the sphere domain and the space surrounding the spheres are stored as separate element sets. Finally, the voxel mesh of the sphere domain is superimposed onto the voxel mesh of the grain microstructure, and the corresponding voxel elements of the former are removed from the latter to recreate the pore geometry in the grain microstructure, as schematically shown in Figure 4c.

### 3.4. Modeling of Pores with Concave Boundaries

**Figure 5:**
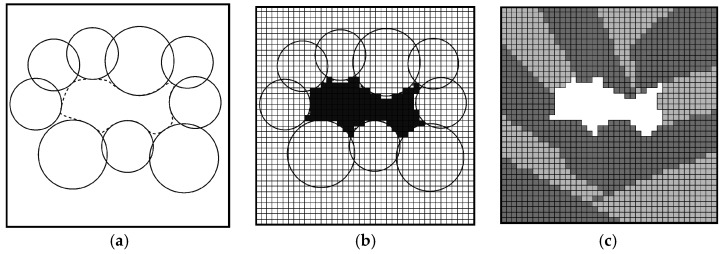
Modeling of pores with concave boundaries (schematic): (**a**) Approximation of the pore geometry (dashed line) by placing overlapping spheres along the pore perimeter; (**b**) Grid discretization of the inter-sphere domain; (**c**) Elimination of the corresponding elements from the voxel mesh of the grain microstructure.

The modeling of pore geometries with concave boundaries differs slightly from the aforementioned procedure: overlapping spheres are again placed strategically within a predefined bounding box. In order to recreate a concave pore boundary, as found in elongated pores caused by a lack of laser energy density, the spheres are arranged along the perimeter of the pore to be modeled. The inter-sphere domain, i.e., the discretized region between the spheres, then forms the final pore geometry, as illustrated in Figure 5a.

In analogy to the procedure for the modeling of pores with convex boundaries, pore shape and orientation within the bounding box can be arbitrarily selected. Voxel discretization of the inter-sphere domain (Figure 5b) and mesh superpositioning then lead to the microstructure model shown in Figure 5c.

## 4. Results

### 4.1. Finite-Element Analyses

In order to illustrate the applicability of the outlined modeling strategy, finite-element analyses on generic, three-dimensional microstructure models of the grains with acicular phases and embedded pores were carried out.

The grain microstructure was generated as described in Section 3.2. To reduce the model size, only six grains were modeled, keeping the grain length/width ratio > 2.5, and only one layer of grains was modeled in the thickness direction. The dimensions of the resulting polycrystal model correspond to 3 × 3 × 3 mm^3^.

Two exemplary pore geometries, one with convex and the other with concave boundaries, were created using the approach described in Section 3.3 and Section 3.4. The pores were generated inside a cubic bounding box with a 2 mm edge length, which is barely larger than the pores themselves. Scaling down the models to a µm-scale will give the same results, as long as the units in which the material properties are specified are consistent.

**Figure 6:**
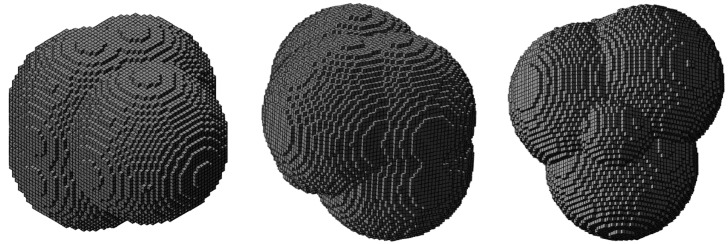
Three views of the exemplary model of a pore with convex boundaries.

**Figure 7:**
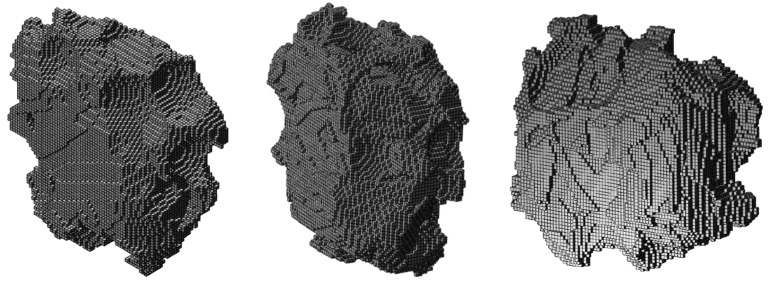
Three views of the exemplary model of an elongated pore with concave boundaries.

For the voxel discretization, voxels with an edge length of 25 µm, which corresponds to 1/80th of the length of the cubic bounding box, were selected due to computational restrictions. Figure 6 and Figure 7 contain detailed views of the generated pore geometries. The pore with a mostly convex boundary, which represents the characteristic geometry of pores formed due to vaporization of the material under an excessive energy density, exhibits predominantly round surfaces, with arbitrary spherical sections (Figure 6).

On the other hand, the pore with a concave boundary, which resembles the pore geometry typical for low energy density, shows tortuous surfaces and many protruding ridges (Figure 7).

The generated pore models were characterized and compared with microanalytical data obtained from actual pore geometries [4]. Both models exhibit an almost identical pore volume *V*. The net pore surface area *A* was determined by dividing the sum of the actual voxel element’s surface area by $\sqrt{2}$ to account for the excessive surface area fraction caused by the jagged pore boundaries. The corresponding values of *A* and the resulting sphericity *ψ* of the exemplary pore geometries are listed in Table 1. As expected, the pore with a convex boundary has a higher sphericity than the elongated pore with a concave boundary.

**Figure 8:**
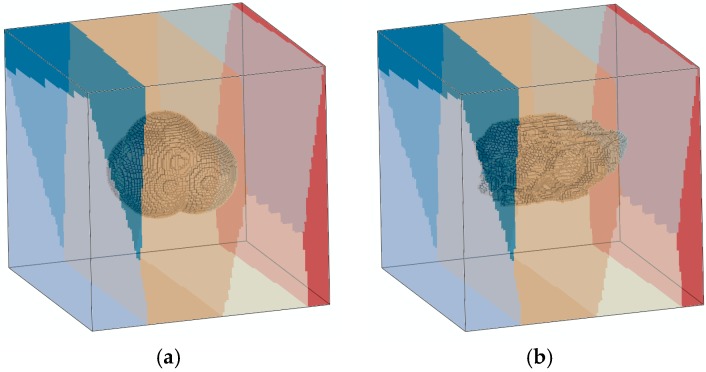
Exemplary virtual microstructure models of grains with embedded pore: (**a**) Round pore with convex boundary; (**b**) Elongated pore with concave boundary. Different colors indicate individual grains.

The equivalent models for the Ti-6Al-4V microstructure associated with high or low energy density during SLM are obtained using superpositioning of the sphere mesh on the grain microstructure mesh, removing the elements of the sphere domain from the grain microstructure mesh. For the exemplary finite-element analyses, only a single pore is embedded in each model. The equivalent virtual microstructure models containing convex and concave pores are shown in Figure 8.

### 4.2. Effect of Pore Morphology on Stress Distribution

Exemplary finite-element analyses of the virtual microstructure models were performed, in order to demonstrate the influence of pore geometry on the local stress evolution. The bulk material of the elongated grains was assumed to follow von Mises plasticity, using the material parameters derived from the mechanical testing of an as-built SLM-processed Ti-6Al-4V alloy published by Kasperovich and Hausmann [23]. The derived elastic modulus *E*_mod_ varies from 99 to 121.6 GPa, and the yield limit *σ*_y_ ranges from 664 to 802 MPa. The Poisson’s ratio *υ* of the grains was assumed to be constant at 0.342, based on the work of Simonelli et al. [35].

To capture the effects of heterogeneity in the grain microstructure, the input material properties of the grains were varied within the upper and lower limit of the elastic modulus *E*_mod_ and the yield point *σ*_y_. This assumption is reasonable, as the martensitic lamellar phases are anisotropic and vary from grain to grain. A more accurate description of the constitutive behavior of the grain microstructure, using a crystal plasticity model, is possible; however, due to the lack of experimental data, such an approach has not been undertaken. The virtual microstructure models were subjected to uniform displacement, prescribed at the top surface. Although the microstructure is not fully symmetric, symmetry boundary conditions were used to avoid strong deformation constraints at the boundaries.

**Figure 9:**
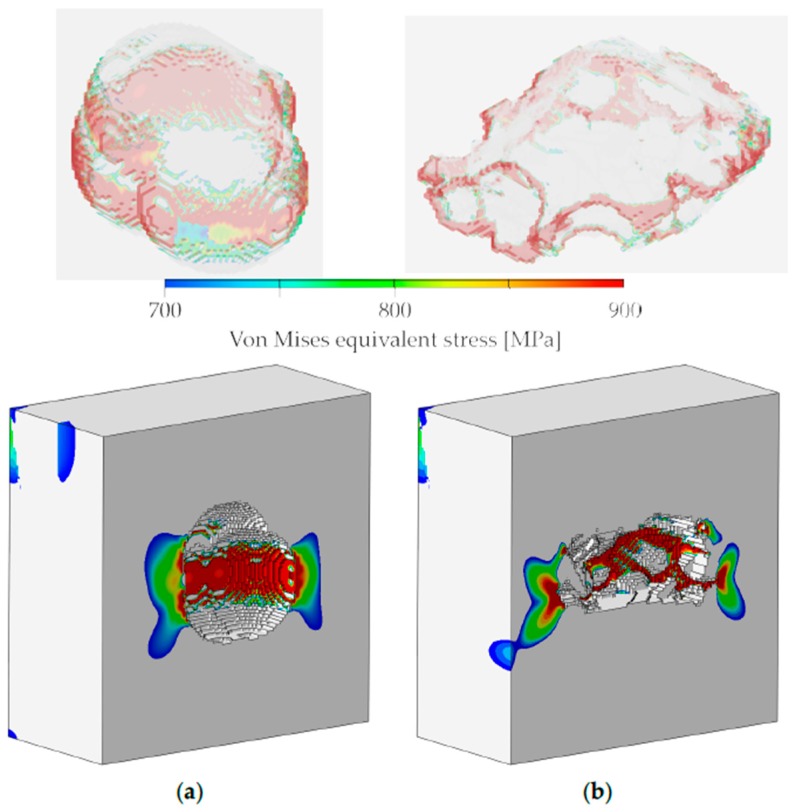
Translucent renderings of the pore domains and cut sections of the analyzed microstructure models with von Mises equivalent stress profiles, illustrating the inhomogeneous distribution of stresses in the vicinity of the embedded pores: (**a**) Circumferential stress profile around the pore with a convex boundary; (**b**) Multiple stress concentrations at the pore with a concave boundary.

After the finite-element analyses, the stress evolution within both microstructure models was compared at approximately 0.2% global strain, to study the effect of pore morphology on local stress concentrations. Figure 9 shows the pore domains, as well as cut sections of the virtual microstructure models, with the von Mises equivalent stress distribution in the vicinity of the embedded pores beyond the average yield limit *σ*_y_ of two-thirds of the grains, which is close to 700 MPa. It is obvious that the stress distribution around the pore with a convex boundary is more gradual and homogeneous, exhibiting a larger plastic zone (Figure 9a), similar to that typically observed in “round-notch”-type specimens. The sharp edges around the pore with a concave boundary lead to an inhomogeneous stress distribution with multiple, narrow plastic zones. The sharp edges act as notches that cause high, localized stress concentrations (Figure 9b), rendering this pore geometry prone to crack initiation. The “butterfly wing” stress contours at the small plastic zones resemble the stress distribution typically found in “sharp-notch” specimens.

The “sharp-notch”-type edges around the pore with a concave boundary may be critical to crack initiation; however, cracks may of course initiate at any location of the narrow yield zones where a comparably high stress concentration evolves.

## 5. Discussion

A strategy for the modeling of the as-built microstructure of a SLM-processed Ti-6Al-4V alloy has been demonstrated, incorporating two essential microstructural features, i.e., elongated grains containing acicular phases and processing-induced pores with a complex geometry. Typical modeling approaches described in the literature often lack an appropriate implementation of these essential features for SLM-processed materials. Recently, digital image-based modeling approaches have been proposed, where the microstructure models are derived directly from volume reconstructions of images obtained by scanning electron microscopy or computed microtomography (µ-CT) [14,16,36,37,38]. Such approaches generally require considerable image manipulation and mesh generation efforts, to obtain microstructure models that are suitable for finite-element analyses.

As an alternative to digital image-based modeling approaches, the outlined feature-dependent modeling approach allows users to make efficient use of different algorithms, in order to recreate the distinct microstructural characteristics common to a Ti-6Al-4V alloy processed by SLM. With parameterized algorithms, a wide range of virtual grain morphologies or pore geometries can be generated and used to evaluate their effect on the effective mechanical properties. Generic microstructure models of a SLM-processed Ti-6Al-4V alloy can be designed “from scratch”. In addition, statistical information derived from microstructural investigations on actual SLM-processed material can be conveniently incorporated during model generation.

In the proposed modeling strategy, the discretization complexity of the microstructure models is minimized and the mesh compatibility is assured by reverting to voxel-based meshing with equal-sized elements. Voxel meshes are easy to generate as they use regular cubic elements. Furthermore, voxel-based meshing guarantees mesh periodicity for periodic microstructure models, rendering it possible to apply periodic boundary conditions. However, the major disadvantages of voxel-based meshing are the lack of discretization accuracy and the occurrence of jagged, “stair-stepped” contours that do not conform to the geometric boundary of the object to be meshed. Due to these steps in the voxel mesh, local stress concentrations and discontinuities may arise between adjacent elements at the domain boundary. The discretization accuracy and the continuity of the stress field can obviously be improved by using very fine voxel meshes with small elements, provided that sufficient computational resources are available. In addition, several boundary smoothing approaches have been developed, to reduce the artifacts common to voxel-based meshing [39,40,41]. One established approach is to trace the domain boundary voxel elements and split them into tetrahedral elements [42]. Other approaches rely on local mesh refinement and enrichment functions [43], or higher-order basis functions [44]. Using such approaches, it is generally possible to improve the representation of pore boundaries after generating the microstructure model using the described mesh superposition technique.

Applying the proposed modeling strategy, exemplary microstructure models of SLM-processed Ti-6Al-4V containing generic pore geometries were generated, and subjected to finite-element analyses. The microstructure models were deformed, assuming elastic-plastic material behavior, to demonstrate the influence of the complex pore geometry on the local stress evolution. Stress concentrations were identified for pores with convex and concave boundaries, to illustrate the morphological effects on local yielding and stress distribution. Such stress concentrations play an important role in the assessment of the fatigue life of additively-manufactured components.

The finite-element analyses of the exemplary microstructure models are not intended to found an in-depth discussion on the effect of pore morphology on the local stress quantities. Nevertheless, the analyses are useful to illustrate how the interdependency between the process parameters, microstructure, and resulting mechanical properties, can be investigated in a finite-element-based simulation framework.

It should be noted that, due to the complexity of the physical processes that interact during the SLM process, an analytical deduction of clear process-microstructure relations is challenging. A common alternative is to empirically investigate the microstructure of material specimens treated under different process parameter settings, and use statistical information derived from such microstructural investigations in order to identify trends on how specific process parameters affect microstructure formation. Extensive experimental and microanalytical investigations need to be conducted to achieve this goal. For the present analysis, the process-microstructure relationship is known a priori, from extensive experimental and microanalytical investigations, as summarized in Refs. [4,23].

The microstructure models generated via the proposed modeling strategy can also be used for determining microstructure-sensitive effective material properties. Combining the relevant microstructural characteristics of SLM-processed Ti-6Al-4V in one numerical model makes it possible to apply single-step homogenization to evaluate the effect of phase properties, pore volume content, or grain orientation, on the overall material properties, e.g., effective elastic moduli or effective conductivity.

The proposed approach of virtual modeling and exemplary stress analysis can potentially be used to correlate the processing, microstructure, and property, with measurable quantities. The predictive capability can be improved by implementing advanced constitutive models to the micromechanical analysis of SLM microstructures, followed by proper validation steps using advanced experiments considering different loading conditions. Such in-depth analyses are beyond the scope of the present work.

## 6. Summary

In this work, a comprehensive modeling strategy for the generation of virtual microstructure models with complex-shaped pores, as found in a SLM-processed Ti-6Al-4V alloy, has been presented. Moreover, a discretization approach of these virtual microstructure models for finite-element analyses has been proposed. The main features of the modeling strategy can be summarized as follows:

(1) The polycrystalline microstructure, consisting of elongated grains with acicular phases typical for Ti-6Al-4V processed via SLM with different process parameters, can be modeled by manipulating the seed generation and Voronoi tessellation within the software tool Neper [34]. A control script has been developed to achieve virtual microstructures that contain the relevant characteristic details of actual material specimens.
(2) Processing-induced pores with complex geometries can be approximated by strategically arranging (partly) overlapping spheres of varied sizes in a predefined bounding box. Statistical equivalence of the morphological characteristics of such virtual pores can be conveniently evaluated and compared with data obtained by microanalytical analyses of actual material specimens. Voxel discretization via a self-written script makes it possible to export the pore geometry to any finite-element software.
(3) A mesh superposition technique has been used in order to combine two or more microstructure models. For a successful application of this superposition technique, all microstructure models need to be discretized with preferably fine voxels, using an equal voxel size for all models. The technique, which is particularly suitable for microstructure models with a complex geometry, has been successfully applied to models representing the microstructure of the SLM-processed Ti-6Al-4V alloy.
(4) The outlined modeling strategy is capable of taking into account microstructure statistics of the grains (grain size distribution, grain orientation, and arrangement of the acicular phases) and pores (pore volume content, spatial distribution of pores, pore size distribution, and pore orientation).
(5) The virtual microstructure models generated via the proposed modeling strategy can be applied to investigate the correlation between the mechanical properties and process parameters of an additively-manufactured Ti-6Al-4V alloy. In addition, the microstructure models can be used to predict effective mechanical properties, using homogenization principles. The damage behavior in the presence of pores and crack-like voids can also be studied, to identify critical pore sizes and shapes with regard to material failure.
(6) The modeling strategy has been exemplarily applied to generate different pore geometries commonly found in a SLM-processed Ti-6Al-4V alloy for different process parameters. Due to generalities of the modeling strategy, it can also be used to model other types of porous metallic microstructures.

## Figures and Tables

**Table 1:** Summary of the geometric descriptors for the generated pore geometries.

Exemplary Pore Geometry	Net Pore Surface Area *A* (mm^2^)	Pore Volume *V* (mm^3^)	Sphericity *ψ*
convex boundary	6.2 ^1^	1.2	0.83
concave boundary	9.9 ^1^	1.2	0.41

^1^ Sum of voxel element surface area divided by 1 Sum of voxel element surface area divided by $\sqrt{2}$.
